# The Mortality of Surgical Operations in the Upper Lake States, Compared with That of Other Regions

**Published:** 1877-02

**Authors:** Edmund Andrews

**Affiliations:** Professor of Princples and Practice of Surgery in Chicago Medical College, Chicago, No. 6 Sixteenth St.


### THE MORTALITY OF SURGICAL OPERATIONS IN THE UPPER LAKE STATES, COMPARED WITH THAT OF OTHER REGIONS.

By EDMUND ANDREWS, A.M., M.D.,

Professor of Princples and Practice of Surgery in Chicago Medical College,

Assisted by Thos. B. Lacey, M.D.,

Assistant Surgeon in the National Soldiers' Home.

(Concluded.)

### OVARIOTOMY.

My records of this operation are mostly from Prof. Byford, and Dr. Dunlap, of Springfield, Ohio.

### TABLE XVI.

### CASES OF OVARIOTOMY PERFORMED BY PROF. W. H. BYFORD.

My first twenty-five operations were performed before the Great Fire, and the notes of them were burned up; the most I can say of them was that the proportion of recoveries was 66⅔ per cent. One of these cases was in the Hospital for Women and Children, and recovered without any bad symptoms. My next thirteen cases are as follows:

| Age. | DURATION OF TUMOR. | SIZE AND DESCRIPTION OF TUMOR. | MARRI'D OR SINGLE. | GENERAL CONDITION BEFORE OPERATION. | MODE OR PECULIARITY OF OPERATION. | RESULT. | TIME TO DEATH OR RECOVERY. | PRACTICE. |
|---|---|---|---|---|---|---|---|---|
| 36 | 3 years | Wt. 37 lbs. Multilocular. Right ovary. Contents of main sac thick as jelly | Married. | Greatly impaired | Secured pedicle with ligature left in wound | Recovered. | 3 weeks | Private |
| 31 | 14 months. | Multilocular. 28 lbs. | Married. | Greatly impaired | Right ovary seat of tumor; left diseased and remov'd | Recovered. | 6 weeks | -------- |
| 32 | 6 years | Both ovaries diseased alike. Two ovarian tumors weighing 20 lbs. | Single. | Greatly impaired | Both ovaries removed | Recovered. | 2 months | Hospit. |
| 42 | 7 months | Two large tumors, one springing from each ovary. Weight, 28 lbs. | Single. | Greatly impaired | Both ovaries removed | Died | 21 days | Private |
| 31 | 9 months | Dermoid tumor, weighing 25 lbs. Left ovary. | Married. | Health poor | -------- | Recovered. | 3 weeks | Private |
| 33 | 1 year | Multilocular of right ovary. Left so diseased as to require removal. Weight, 37 lbs. | Married. | Health good | -------- | Recovered. | 5 weeks | -------- |
| 23 | 1 year | Multilocular. 34 lbs. | Single. | V ry much impar | -------- | Recovered. | 3 weeks | -------- |
| 36 | 7 years | Multilocular. 28 lbs. | Married. | Very much impar | -------- | Died | 14 days | -------- |
| 18 | 18 months. | Dermoid cyst. 27 lbs | Single. | Health good | -------- | Recovered. | 2 weeks | -------- |
| 24 | 9 years | Multilocular. 30 lbs. | Single. | Very much impar | -------- | Recovered. | 2 months | -------- |
| 24 | 17 months. | Multilocular. 15 lbs. | Married. | Very much impar | -------- | Recovered. | 2 weeks | -------- |
| 43 | 11 months. | Dermoid. 30 lbs. | Married. | Good condition | -------- | Recovered. | 4 weeks | -------- |
| 27 | 3 years | Multilocular. 45 lbs. | Married. | Emaciated | -------- | Recovered. | 6 weeks | -------- |

In addition to the above I have accounts of 118 cases, of which 34 died, operated on by the following surgeons, viz.: Dr. Dunlap, of Springfield, Ohio, and Drs. E. Andrews, J. Andrews, D. Brainard, Brauns, A. Fisher, J. M. Hutchinson, A. R. Jackson and E. O. F. Roler.

RECAPITULATION.

| AUTHORITIES. | CASES. | DIED. |
|---|---|---|
| Byford's operations before the great fire (notes burned up) | 25 | 8 |
| Byford's operations since | 13 | 2 |
| Dunlap's operations | 107 | 26 |
| Other Lake State operators | 11 | 8 |
| Total Lake States cases | 156 | 44 |

Mortality in the Lake States, 28 per cent.

OVARIOTOMY ABROAD.

| AUTHORITIES. | CASES. | DIED. |
|---|---|---|
| W. L. Atlee, Philadelphia | 350 | 105 |
| Prof. Peaslee, N. Y. | 28 | 9 |
| Dr. G. Kimball, of Lowell, Mass. | 203 | 67 |
| Spencer Wells, of England, 1876 | 500 | 127 |
| Dr. Clay, of Eng., quoted in Peaslee on Ov. Tum., p. 248 | 250 | 68 |
| Dr. Keith of Scotland, " " " " | 136 | 25 |
| Thomas " " " " | 27 | 9 |
| Bradford " " " " | 30 | 3 |
| Dr. Cheever, of Boston | 4 | 4 |
| Bryant (Trans. Obst. Soc., London, 1865) | 10 | 4 |
| Tyler Smith, " " " | 20 | 5 |
| St. Thomas' Hosp. | 3 | 1 |
| St. Bartholomew's Hosp | 23 | 17 |
| St. George's Hosp. | 5 | 4 |
| Grimsdale, quoted Arch. klin. Chir. Bd. 8, S. 813 | 10 | 3 |
| Cases in Germany, Russia, Switzerland, Italy, Spain, Australia and India, Arch. klin. Chir. Bd. 8 | 35 | 15 |
| K. k. allg. Krankenhaus, Wien | 25 | 17 |
| Roosevelt Hosp. | 1 | 1 |
| Totals | 1660 | 484 |

Mortality, 29 per cent.

GENERAL SUMMARY.

| | CASES. | DIED. | PER CENT. MORTLI'Y. |
|---|---|---|---|
| Lake States | 156 | 44 | 28 |
| Abroad | 1660 | 484 | 29 |

OPINIONS OF AUTHORS.

In the treatment of ovarian cysts two operations have to be considered, viz.: Tapping and ovariotomy. On these points Prof. W. H. Byford, of Chicago, has favored me with the following opinions:

"TAPPING."

"Tapping an ovarian tumor is always attended with danger, and ought not to be resorted to without important reasons. This operation is especially hazardous in the polycystic variety.

"It is allowable in monocysts, when the diagnosis is doubtful, for the purpose of deciding the nature of the fluctuating mass.

"When the collection of fluid is very great and the patient in an exhausted condition, by evacuating it the patient will generally recruit under proper treatment. She will then bear ovariotomy better.

"If for any reason ovariotomy is impracticable, we may often palliate the suffering and prolong the life of the patient by tapping one or more times, as the case may require.

"Again, there is another condition, not very rare, in which tapping may be relied upon as curative, *i. e.*, when the vitality of the tumor is decreasing. This condition is more frequently observed in patients somewhat advanced in years, and is recognizable by what I would denominate tentative tapping, or the history of the case connected with this operation. If after several evacuations the length of time in which the tumor fills up is increasing, we may expect by repetition of the operations the vitality of the growth will be exhausted and eventually will not fill again. I have seen two remarkable instances of this kind, in which the patients recovered after they had been tapped a number of times."

"INDICATIONS FOR OVARIOTOMY."

"We are justified in the performance of ovariotomy only when the patient's health is becoming impaired in consequence of the presence of the tumor. This will occur when it is large enough to press mischievously upon the vital organs. Of course other indications, under special circumstances, may determine the propriety of the operation, but it would not be expedient here to enter upon the consideration of them, as it would require too much space.

"Ovariotomy should not be thought of until the diagnosis is so clearly demonstrated as to leave no doubt in the mind of the operator."

Mr. Bryant, surgeon to Guy's hospital, thinks that tapping should be omitted in the majority of cases, unless needed for the purpose of diagnosis. Spencer Wells, however, whose vast experience gives weight to his opinion, thinks that previous tapping does not materially affect the safety of a subsequent ovariotomy.

Mr. Bryant thinks that ovariotomy should be performed in almost all cases of benign polycystic ovarian tumor, except when the patient's health is so broken down as to render it nearly certain that she will not bear the operation. As to the time to be selected he thinks ovariotomy should not be thought of until the health of the patient begins to suffer seriously from the growth of the tumor.

Jonathan Hutchinson discourages mere tapping, but speaks favorably of injections of iodine in the few unilocular cases. He favors ovariotomy strongly in proper cases, and reckons the risk at about 33 per cent.

Spencer Wells, and all the other great ovariotomists, of course favor the operation in proper cases, and it is scarcely worth while to quote against their decisive authority the crude objections of less experienced men in the earlier years of the discussion of this subject.

### CONCLUSIONS.

The mortality of this operation, in the Lake States, has been 28 per cent. for all cases collected by me, but only 26 per cent. in the hands of Prof. Byford. Abroad the mortality has been 29 per cent., but grows somewhat less as skill and experience accumulate. The operation has been safer in Great Britain than on the continent of Europe.

It is a grave operation and never to be undertaken except after full investigation of each case, but there is no doubt that it is firmly established as one of the great operations of surgery. As remarked by Prof. Byford, it ought not to be performed until the patient's health begins to suffer from the pressure of the tumor, and a very careful investigation of all the conditions of the case should be made before decision; and then, if it is found that no insuperable obstacle exists, the operation is to be positively recommended, for after the tumor begins to interfere with the functions of vital organs, the short and miserable remnant of a life, without an operation, may rationally be risked for two chances out of three for a permanent cure.

With regard to tapping the tumor, nothing better can be said than the judicious words of Prof. Byford, quoted above under the head of "Opinions of Authors."

### TRANSFUSION.

This operation has been performed in the Lake States but few times, so far as I can learn. The records are very imperfect, but some interesting observations have been made. Prof. Freer and Prof. E. Andrews have transfused for hæmorrhage in eight or ten cases. Prof. Freer's cases were the most numerous of the two, and one of them was so greatly improved as to give the highest hopes of recovery, when the patient suddenly died with symptoms of embolism. None of the cases of either operator finally recovered. Dr. Hotz transfused one case of hæmorrhage with lamb's blood, with the result of saving the patient. Dr. Hotz, together with Dr. Prœgler and Dr. Wild, transfused, with lamb's blood, in eight cases of phthisis and anæmia. One was temporarily improved and one died of the effects of the operation. Dr. Hotz is of the opinion that the operation should be limited to cases of recent hæmorrhage, but Prof. Freer is disposed to think that if Dr. H. had repeated the transfusion some of the failures might have been transformed into successes.

Prof. Freer has experimented largely on dogs, and from his observations concludes that the transfusion of defibrinated blood is the most successful plan.

Abroad the operation, if honestly quoted, has been more successful. Laudois gives 96 finished cases, of which only 31 died, being a mortality of 32 per cent.

The opinions of authors on this operation are generally expressed in rather vague terms, but for the most part they favor it for desperate hæmorrhage.

Gross, Freer, Ashurst, Moore, of England, Blundell and others favor it decidedly. Freer and Ashurst prefer defibrinated blood.

### CONCLUSIONS.

The literature of transfusion is still in a very crude condition. My opinion is, however, that in cases of dangerous hæmorrhage it is an important resource, and that it is best performed with defibrinated blood. I think the ill success which has attended it in the Lake States is due to the reluctance of surgeons to undertake it promptly, and to the consequent fact that the patients were generally too far gone for recovery.

### MISCELLANEOUS OPERATIONS IN THE LAKE STATES.

The following list contains a number of scattered cases recorded by myself and others worthy, perhaps, of notice, but not numerous enough to be tabulated in detail:

| OPERATIONS. | CASES. | FAILED | DIED. |
|---|---|---|---|
| Trephining for frac of skull | 10 | 0 | 3 |
| " for insanity after fracture | 2 | 1 | 1 |
| " for idiopathic insanity | 2 | 2 | 0 |
| " for epilepsy (permanancy of successes not known) | 7 | 1 | 1 |
| Ligation of common carotid artery for traumatic hæmorrhage | 1 | 0 | 0 |
| Ligation com. carotid for vascular tumor of orbit | 1 | 0 | 0 |
| Ligation of brachial art. for traumatic aneurism | 1 | 0 | 0 |
| Ligation of common iliac art. for aneurism of aorta | 1 | 0 | 1 |
| Ligation ext. iliac for traumatic aneurism | 1 | 0 | 0 |
| Ligation of ext. iliac for aneurism of femeral | 1 | 0 | 0 |
| Ligation of femeral for wounds | 3 | 0 | 0 |
| " " " aneurism | 2 | 1 | 0 |
| Compression of arteries for aneurism* | 6 | 4 | |
| Stricture of uretha treated by internal section* | 24 | 1 | 0 |
| " " " divulsion* | 15 | 2 | 0 |
| " " " external perin sect.* | 2 | 0 | 0 |
| " " " gradual dilatation* | 50 | 0 | 0 |
| Operations for hæmorrhoids* | 45 | 0 | 0 |
| Forcible rupture of anchylosis† | 8 | 3 | 1 |
| Operations for ununited fracture* | 24 | 1 | 0 |
| Stretching sciatic nerve for neuralgia (Nussbaum's operation) | 2 | 0 | 0 |
| Neurotomy | 8 | 0 | 0 |

* Records very imperfect.
† Three of the successes were imperfect.

For convenience of reference to those who wish to see at a glance what the experience of the world has been with regard to the principal operations, the following table is prepared:

### TABLE XVII.

### MORTALITY OF THE PRINCIPAL OPERATIONS.

| CASES. | PER CENT. LAKE STATES. | PER CT. ABROAD. |
|---|---|---|
| Amputation at shoulder joint, primary | | 35 |
| " " " second. and inter. comb. | | 48 |
| " " " pathological | | 29 |
| " " " average of all cases | 30 | 39 |
| " arm, primary | 20 | 27 |
| " " intermediary and second. combin. | | 36 |
| " " pathological | | 20 |
| " " average of all cases | 11 | 35 |
| " elbow joint, average of all cases | | 21 |
| " forearm, primary | | 11 |
| " " intermediary | | 23 |
| " " secondary | | 16 |

TABLE XVII.—Continued.

| | | |
|---|---|---|
| Amputation at forearm, pathological | | 22 |
| " " average of all cases | 10 | 16 |
| " wrist, primary | | 8 |
| " " intermediary | | 14 |
| " " secondary | | 20 |
| " " pathological | | 7 |
| " " average of all cases | | 18 |
| " hip joint, primary | | 99 |
| " " " intermediary | | 92 |
| " " " secondary | | 65 |
| " " " pathological | | 47 |
| " " " average of all cases* | 43 | 30 |
| " upper 3d of thigh, primary | 40 | 60 |
| " " " " inter. and sec. comb'd | | 44 |
| " " " " pathological | | 27 |
| " middle " " primary | | 48 |
| " " " " inter. and sec. comb'd | | 59 |
| " " " " pathological | | 22 |
| " lower " " primary | 28 | 45 |
| " " " " inter. and sec. comb'd | | 60 |
| " " " " pathological | | 20 |
| " average of all thigh cases | 24 | 62 |
| " knee joint, primary | | 48 |
| " " " intermediary and sec. comb'd | | 49 |
| " " " pathological | | 26 |
| " " " details not stated | | 54 |
| " leg, upper 3d, primary | | 36 |
| " " " " intermed. and sec'y comb'd | | 47 |
| " " " " pathological | | 31 |
| " " " " average of all cases | 36 | 37 |
| " " middle " primary | | 45 |
| " " " " intermed. and secondary | | 44 |
| " " " " pathological† | | 6 |
| " " " " average of all cases | 25 | 32 |
| " " lower " primary | | 37 |
| " " " " intermed. and sec'y comb'd | | 29 |
| " " " " pathological | | 16 |
| " " average of all cases | 13 | |
| " " average of all times and locations | 23 | 40 |
| " ankle, Syme's | | 9 |
| " " Pirogoff's | 22 | 18 |
| " foot, Chopart's | | 16 |
| Resection, shoulder, primary | | 30 |
| " " intermediary | | 46 |
| " " intermed. and second. together | | 37 |
| " " pathological | | 19 |
| " " average of all cases | | 32 |
| " elbow, primary | | 22 |
| " " intermediary | | 35 |
| " " intermed. and second. combined | | 28 |
| " " secondary, alone | | 9 |
| " " pathological | | 12 |
| " wrist, average of all cases | | 19 |
| " hip, primary | | 92 |
| " " intermediary | | 91 |

* This per cent. in the Lake States was derived from only seven cases. It cannot be expected to continue so low in the future.

† Derived from forty-nine cases. Mortality accidentally low.

TABLE XVII.—Continued.

| | | |
|---|---|---|
| Resection, hip, intermed. and second. combined | | 71 |
| " " secondary | | 85 |
| " " pathological | 42 | 46 |
| " knee, all traumatic combined | | 85 |
| " " pathological | 37 | 29 |
| " ankle, all traumatic combined | | 13 |
| " " pathological | 11 | 12 |
| Herniotomy | 24 | 49 |
| Hernia, radical cure operations | | 4 |
| Lithotomy, 1 to 5 years | | 7 |
| " 5 to 11 years | | 4 |
| " 11 to 16 years | | 11 |
| " 16 to 20 years | | 14 |
| " 20 to 30 years | | 13 |
| " 30 to 38 years | | 9 |
| " 38 to 48 years | | 17 |
| " 48 to 58 years | | 21 |
| " 58 to 70 years | | 27 |
| " 70 to 80 years | | 32 |
| " average under puberty | | 7 |
| " " over " | 38 | 19 |
| " " all ages combined | 23 | 16 |
| Lithotrity | | 9 |
| Tracheotomy and laryngotomy for diphtheria & croup, | | |
| Under 2 years | | 100 |
| 2 to 3 years | | 73 |
| 3 to 4 years | | 68 |
| 4 to 5 years | | 76 |
| 5 to 6 years | | 65 |
| 6 to 7 years | | 55 |
| 7 to 8 years | | 76 |
| 8 to 14 years | | 58 |
| All ages together | 81 | 63 |
| Tracheotomy and laryngotomy for œdema glottidis | 25 | 26 |
| " " for foreign bodies | | 25 |
| Œsophagotomy | | 24 |
| Ovariotomy | 28 | 29 |
| Transfusion for hæmorrhage | | 32 |
| Ligation of the aorta | | 100 |
| " common iliac artery | | 76 |
| " internal " " | | 58 |
| " external " " | | 43 |
| " femoral " " | | 34 |
| " profun. femoris " | | 62 |
| " popliteal " | | 76 |
| " arteries of leg and foot | | 50 |
| " innominata artery | | 94 |
| " common carotid " | | 45 |
| " internal " " | | 12 |
| " subclavian " | | 55 |
| " axillary " | | 75 |
| " brachial " | | 19 |
| " radial and ulnar " | | 15 |
| Trephining for fracture of cranium | | 50 |
| " for epilepsy | | 58 |
| Colotomy | | 44 |
| Gastrotomy for stricture, foreign bodies, etc. | | 58 |
| Extraction of loose cartilages from the knee | | 20 |

I have in this unusually prolonged article endeavored to give a condensed view of the statistics and opinions of the world, on every principal operation in surgery. It has been a work of immense labor, but yet a very necessary one, for the contradictions of authors and the very frequent hastiness and superficiality displayed in writings of recognized authority render it almost impossible for the practical surgeon to distinguish truth from error.

I return my thanks to those gentlemen who have contributed their cases for the Lake States lists, and their names will be found in the tables.

I regret that the great Chicago fire swept out of existence the records of several excellent surgeons, thus depriving me of the benefit of their extensive experience; but the cases which I did obtain have been carefully sifted and fairly represent the results of surgery in this region.

CHICAGO, No. 6 Sixteenth St., Dec. 1, 1876.

